# Single-cell and spatial transcriptomic insights into glioma cellular heterogeneity and metabolic adaptations

**DOI:** 10.3389/fimmu.2025.1561388

**Published:** 2025-04-04

**Authors:** Yixin Fu, Yong Yi, Yongxiang Shao, Jingcheng Jiang, Qingshan Deng

**Affiliations:** Department of Neurosurgery, The Second People’s Hospital of Yibin, Yibin, Sichuan, China

**Keywords:** glioma, tumor microenvironment, single-cell, tumor-associated macrophages, metabolic reprogramming

## Abstract

Glioblastoma, one of the most aggressive and heterogeneous malignant tumors, presents significant challenges for clinical management due to its cellular and metabolic complexity. This review integrates recent advancements in single-cell RNA sequencing (scRNA-seq) and spatial transcriptomics to elucidate glioblastoma’s cellular heterogeneity and metabolic reprogramming. Diverse cellular subpopulations, including malignant proliferative cells, stem-like cells, mesenchymal-like cells, and immune-related cells, contribute to tumor progression, treatment resistance, and microenvironmental interactions. Spatial transcriptomics has further revealed distinct spatial distributions of these subpopulations, highlighting differences in metabolic activities between the tumor core and periphery. Key metabolic adaptations, such as enhanced glycolysis, fatty acid oxidation, and glutamine metabolism, play critical roles in supporting tumor growth, immune evasion, and therapeutic resistance. Targeting these metabolic pathways, especially in combination with immunotherapy, represents a promising avenue for glioblastoma treatment. This review emphasizes the importance of integrating single-cell and spatial multi-omics technologies to decode glioblastoma’s metabolic landscape and explore novel therapeutic strategies. By addressing current challenges, such as metabolic redundancy and spatiotemporal dynamics, this work provides insights into advancing precision medicine for glioblastoma.

## 1 Introduction

Gliomas, particularly glioblastomas, are highly aggressive and treatment-resistant malignant tumors. Their heterogeneity presents significant challenges for clinical management. While a combination of surgery, radiotherapy, and chemotherapy can modestly improve patient survival, the overall prognosis remains grim (1, 2). Cellular heterogeneity within gliomas is a key factor contributing to tumor recurrence and treatment resistance. Different cellular subpopulations exhibit marked differences in gene expression, metabolic requirements, and functional states (3, 4). This heterogeneity not only complicates therapeutic approaches but also drives tumor progression. Therefore, a deeper understanding of the characteristics of various cellular populations within the tumor is critical for developing personalized treatment strategies.

In recent years, technological advancements like single-cell RNA sequencing (scRNA-seq) have empowered researchers to conduct detailed gene expression analyses at the single-cell level within the tumor microenvironment, revealing the intricate cellular heterogeneity of tumors (5). Through single-cell sequencing, scientists have identified multiple cellular subpopulations with distinct functional and molecular characteristics in gliomas, including malignant proliferative cell populations, stem-like cell populations, mesenchymal-like cell populations, neuron-like cell populations, and immune-related cell populations (6, 7). These subpopulations are involved in different tumor biological processes; for instance, stem-like cell populations are closely associated with tumor self-renewal and drug resistance, while mesenchymal-like cell populations exhibit high invasiveness (8). These findings reveal the complex structure of gliomas and provide a foundation for identifying more effective therapeutic targets.

Tumor metabolic reprogramming is a critical mechanism that enables tumor cells to adapt and thrive within their microenvironment. Glioma cells exhibit unique metabolic characteristics, including enhanced glycolysis, glutamine dependency, and fatty acid oxidation, which provide them with advantages for rapid growth and survival (9, 10). However, different cellular subpopulations within gliomas exhibit distinct metabolic demands and pathways, further contributing to tumor heterogeneity (10). These metabolic alterations not only influence the behavior of tumor cells themselves but also affect other cells in the tumor microenvironment through metabolic byproducts, such as lactate, which can suppress the activity of immune cells and help tumor cells evade immune clearance (11). Therefore, uncovering the metabolic characteristics of different cellular subpopulations and their interactions within the tumor microenvironment is critical for understanding tumor metabolic reprogramming and developing metabolism-targeted therapies (12).

In summary, this review aims to integrate the application of single-cell sequencing in glioma subpopulation research, providing a detailed analysis of the metabolic characteristics of various glioma cell subpopulations and their roles in the tumor microenvironment. Furthermore, it explores the potential of metabolic reprogramming as a therapeutic target, highlighting the impact of glioma metabolic features on tumor progression and therapeutic responses. This work seeks to offer new insights and directions for future glioma treatment strategies.

## 2 Cellular heterogeneity in gliomas

### 2.1 Discovery of glioma cellular subpopulations

**Figure 1:**
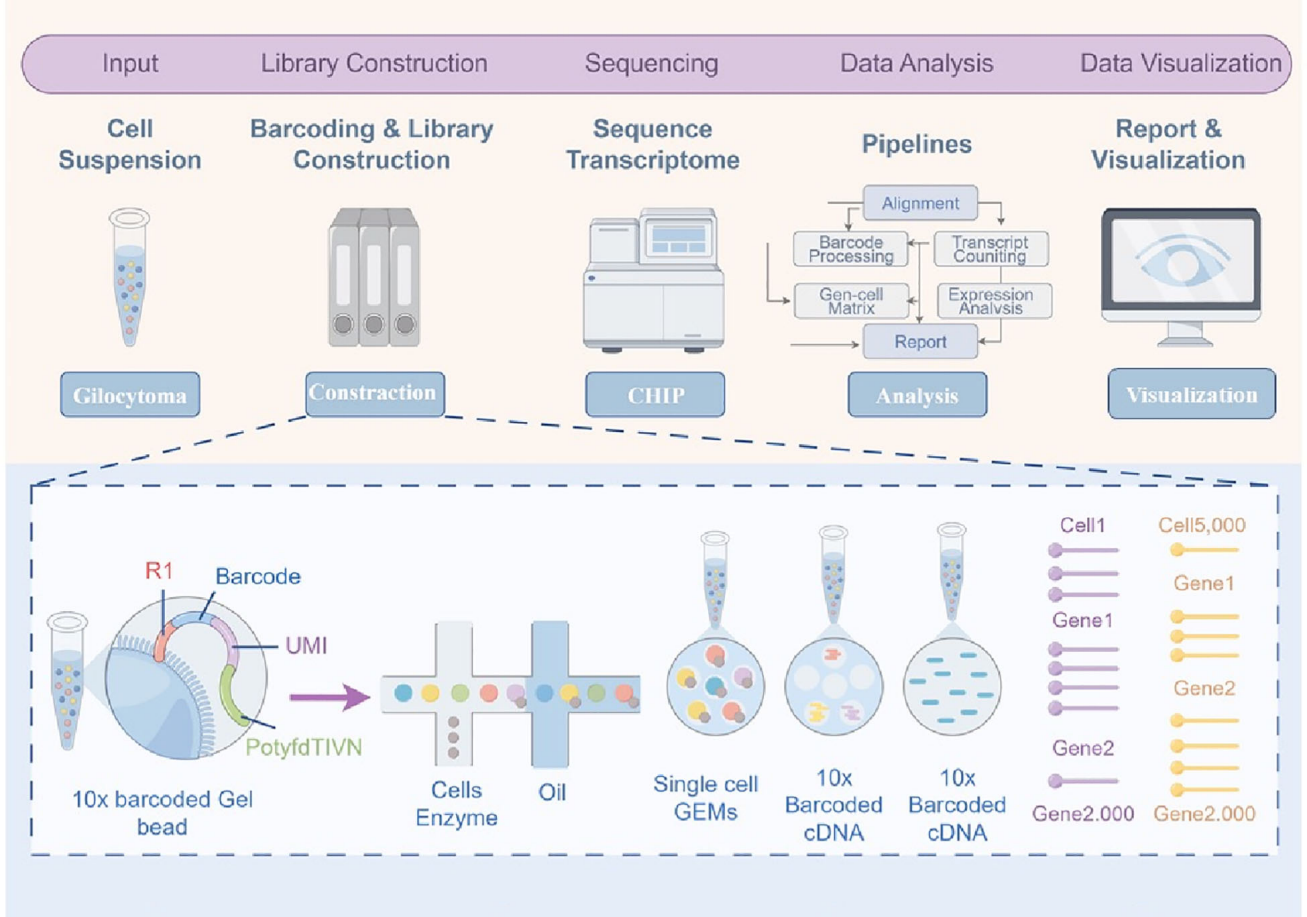
Workflow of single-cell transcriptomics for glioblastoma analysis. This figure illustrates the workflow for single-cell transcriptomics applied to glioblastoma, detailing the process from cell suspension preparation to data visualization. Glioblastoma tissue is first dissociated into a single-cell suspension, serving as the input for the analysis. In the barcoding and library construction step, single cells are encapsulated with 10x barcoded gel beads and enzymes in microfluidic droplets to form Gel Bead-in-EMulsions (GEMs), where each bead carries unique barcodes and unique molecular identifiers (UMIs) to label individual transcripts. Within the droplets, reverse transcription occurs, producing 10x barcoded complementary DNA (cDNA), which is later amplified and sequenced using high-throughput sequencing technologies. The sequencing data is processed through computational pipelines to align reads, process barcodes, count transcripts, and generate a gene-cell expression matrix. Advanced analytical tools are then applied to identify differentially expressed genes, cluster cells, and reveal cellular heterogeneity within the tumor. Finally, data visualization methods are employed to create interpretable outputs, such as heatmaps and clustering plots, which provide insights into glioblastoma cell populations and their molecular characteristics. The inset panel provides a detailed view of the barcoding process, where individual cells, enzymes, and 10x barcoded beads are encapsulated in oil droplets (GEMs), enabling cell-specific transcript capture and accurate reconstruction of gene expression profiles. This workflow highlights the power of single-cell transcriptomics in uncovering glioblastoma heterogeneity and identifying molecular pathways driving tumor progression. GEMs, Gel Bead-in-EMulsions; UMI, Unique Molecular Identifier; cDNA, Complementary DNA.

Gliomas, particularly glioblastoma (GBM), are among the most heterogeneous tumor types known. According to the 2021 WHO classification of central nervous system tumors, gliomas are graded from 1 to 4, with grades 1 and 2 classified as low-grade gliomas and grades 3 and 4 as high-grade gliomas (3, 13). This heterogeneity is observed not only between different patients (inter-tumoral heterogeneity) but also within a single tumor, across different regions and cellular populations (intra-tumoral heterogeneity) (14). Traditional research methods often fail to accurately capture this complexity due to the confounding effects of bulk sample analysis. However, the development of single-cell RNA sequencing (scRNA-seq) has provided a transformative approach to elucidate the cellular subpopulations within gliomas (15)(**Figure 1**). Using scRNA-seq, researchers have identified diverse cellular subpopulations within gliomas that exhibit distinct functional characteristics and gene expression profiles (16). These subpopulations display significant differences in their functional and metabolic traits, which influence tumor invasiveness, therapeutic resistance, and interactions with the tumor microenvironment (17).

Malignant proliferative cell populations serve as the primary drivers of tumor expansion, characterized by the high expression of proliferation markers such as KI-67 and EGFR. These cells often exhibit enhanced signaling through pathways such as EGFR or PDGFRA, which are critical for driving tumor growth (18, 19). Studies have shown that this subpopulation is primarily located within the tumor core, where it exhibits limited adaptability but relies heavily on high metabolic activity to support proliferation (20). Stem-like cell populations, on the other hand, are defined by the high expression of tumor stemness markers such as SOX2, OLIG2, and NESTIN. These cells sustain tumor heterogeneity through their abilities for self-renewal and differentiation and are closely associated with resistance to chemotherapy and radiotherapy. Research indicates that stem-like cells adapt to hypoxic environments by enhancing glycolysis and fatty acid oxidation to meet their energy demands (21). Mesenchymal-like cell populations play a key role in glioma invasion and migration (22). Tumor cells undergo an epithelial-to-mesenchymal transition (EMT), acquiring greater invasive and metastatic potential (23). These mesenchymal-like cells are typically found at the tumor margins, where they are strongly associated with invasion into surrounding tissues and the promotion of angiogenesis (22). Neuron-like cell populations express neuronal differentiation markers such as NEUROD1 and TUBB3. While their specific role in gliomas remains unclear, some studies suggest that these cells may represent an intermediate state of malignant differentiation in gliomas (24, 25). The immune cell component of the glioma microenvironment is primarily composed of tumor-associated macrophages (TAMs), regulatory T cells (Tregs), and a small population of activated effector T cells (26). TAMs display immunosuppressive marker genes and secrete factors such as TGF-β and IL-10, which suppress antitumor immune responses. Tregs, characterized by the high expression of FOXP3, further exacerbate immune evasion by inhibiting the activity of effector T cells. These findings highlight the diverse roles of glioma subpopulations in tumor progression, resistance to therapy, and interaction with the microenvironment. Understanding the functional characteristics of these subpopulations provides a basis for developing targeted therapies aimed at disrupting their contributions to glioma pathophysiology.

Glioma cells exhibit high plasticity, allowing dynamic transitions between different cellular subpopulations under specific conditions (12). For instance, stem-like cells can differentiate into malignant proliferative cells or mesenchymal-like cells. Such subpopulation transitions play a crucial role in tumor adaptation to external pressures, such as therapy or hypoxic conditions (21). Monocyte-derived macrophages are the primary immunosuppressive TAMs in glioblastoma. Recent studies have demonstrated that PERK-mediated glycolysis drives histone lactylation modifications, which enhance the immunosuppressive capabilities of these TAMs (27). The spatial distribution of glioma subpopulations further reflects their functional diversity. Malignant proliferative cells are predominantly localized in the tumor core, whereas mesenchymal-like cells and immune-related cells tend to be found at the tumor margins (28). Stem-like cells are distributed throughout both the core and the periphery, leveraging their differentiation potential to adapt to various microenvironmental conditions.

### 2.2 Spatial transcriptomics in deciphering cellular spatial distribution

The spatial heterogeneity of gliomas is a central driver of their invasiveness, therapeutic resistance, and recurrence (12). In addition to the dynamic characteristics of cellular subpopulations, the spatial distribution patterns of different subpopulations within the tumor core and margins significantly influence the biological behavior of gliomas (29). While traditional scRNA-seq has revealed the gene expression profiles of glioma cells, it lacks spatial information, making it insufficient to fully resolve the physical relationships between cells and their interactions with the tumor microenvironment. ST technology, which simultaneously captures gene expression and spatial location within tissue sections, offers a new perspective for analyzing the spatial heterogeneity of gliomas. In recent years, this technology has been widely applied in glioma research, advancing our understanding of cell-cell interactions within the tumor and the molecular differences between the core and peripheral regions. By integrating spatial information, ST enables a more comprehensive investigation of glioma biology, shedding light on the interactions between tumor cells and their microenvironment, as well as uncovering region-specific molecular characteristics that may serve as potential therapeutic targets (12, 28).

The advantages of spatial transcriptomics lie in its ability to resolve the spatial distribution of the tumor microenvironment. First, it reveals the functional characteristics and gene expression differences between the tumor core and peripheral regions. The tumor core is typically composed of highly proliferative malignant cells that rely on metabolic pathways such as glycolysis to adapt to extreme conditions of hypoxia and nutrient deprivation. Cells in the core region exhibit high levels of hypoxia-inducible factor (HIF-1α) and glycolysis-related genes, while the accumulation of lactate further reinforces an immunosuppressive state, for instance, by promoting the upregulation of immune checkpoint molecules like PD-L1 (30). In contrast, the peripheral tumor region exhibits an immunologically “cold” microenvironment. Within the tumor, damage-associated programs dominate immune infiltration, leading to the emergence of low-proliferative, injury-induced neural progenitor-like cells (iNPCs). Meanwhile, in the immunologically cold invasive tumor margins, cells tend to differentiate into invasive astrocyte-like glioma cells (23). Furthermore, immune cells in the margins are regulated by lactate and lipid molecules, which suppress their functionality and contribute to tumor immune evasion (31).

Spatial transcriptomics also enables the resolution of spatial distribution patterns and dynamic interactions of different cellular subpopulations at the single-cell level. For instance, malignant proliferative cells are predominantly clustered in the tumor core, reflecting their reliance on high metabolic demands and proliferation-driving mechanisms (12). Stem-like cells, due to their multifunctionality and differentiation potential, are widely distributed across both the core and peripheral regions, serving as a major source of glioma heterogeneity (32). Mesenchymal-like cells are concentrated at the tumor margins, where they are closely associated with invasion and angiogenesis, while neuron-like cells are more diffusely distributed, potentially representing an intermediate state of malignant differentiation (12, 33). Immune-related cells, such as TAMs)and regulatory T cells (Tregs), are primarily located in the peripheral region, where they regulate immune balance within the tumor microenvironment. By suppressing the function of effector immune cells, these immune subpopulations create a protective barrier that facilitates tumor spread (34). These spatial characteristics, meticulously revealed through spatial transcriptomics, provide powerful insights into the metabolic and signaling interactions among cellular subpopulations. This technology offers a robust foundation for studying the complex dynamics of tumor microenvironments and exploring new therapeutic strategies targeting these interactions.

In addition to revealing spatial distribution patterns, spatial transcriptomics, combined with metabolic analysis tools such as scFEA and scMetabolism, has uncovered distinct metabolic characteristics between the core and peripheral regions of gliomas and their impact on the tumor microenvironment. In the tumor core, where hypoxia and nutrient deprivation dominate, metabolism primarily shifts toward glycolysis and lactate metabolism (the Warburg effect). In contrast, the peripheral region, being closer to normal tissues and thus more accessible to oxygen and nutrients, exhibits enhanced glutamine metabolism and fatty acid oxidation (35). Moreover, spatial transcriptomics enables the inference of metabolic interactions between different cellular subpopulations. For instance, mesenchymal-like cells regulate the metabolic state of tumor-associated macrophages (TAMs) by secreting chemokines, while stem-like cells modulate the activity of surrounding immune cells through lactate secretion (36). This integrated analysis of metabolism and spatial distribution offers a novel perspective on how the metabolic microenvironment shapes tumor progression. It provides valuable insights into the interplay between metabolism and cellular interactions, paving the way for the development of innovative therapeutic strategies targeting tumor metabolism and its spatially defined microenvironment (37).

In conclusion, spatial transcriptomics provides a revolutionary tool for understanding tumor spatial heterogeneity by resolving the spatial distribution and metabolic characteristics of glioma cells. It not only uncovers the distribution and functional properties of cellular subpopulations across core and peripheral regions but also lays the foundation for precision therapeutic strategies through spatially resolved analyses of metabolic features and cellular interactions. Looking ahead, integrating temporal dynamics into spatial studies and combining spatial transcriptomics with other multi-omics data, such as mass spectrometry imaging, holds great promise for further uncovering the complex biological features of gliomas. These advancements could provide robust support for developing personalized therapeutic strategies targeting different tumor regions, ultimately improving outcomes for glioma patients.

## 3 Metabolic reprogramming in gliomas

### 3.1 Metabolic characteristics of different cellular subpopulations

**Figure 2:**
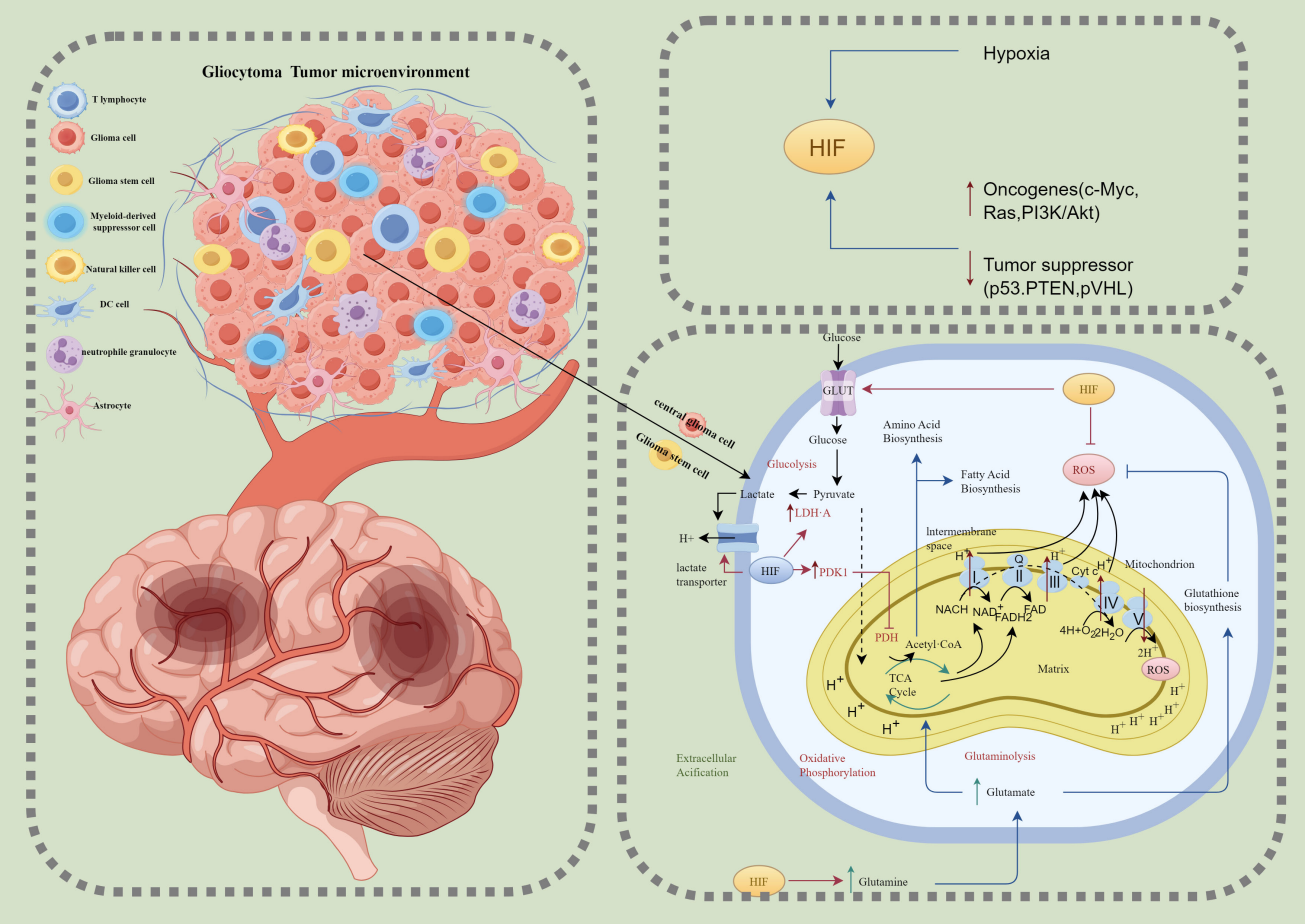
Glioblastoma tumor microenvironment and hypoxia-induced metabolic reprogramming. This figure illustrates the glioblastoma tumor microenvironment (left) and the metabolic adaptations of glioblastoma cells under hypoxic conditions (right). The tumor microenvironment comprises various cell types, including T lymphocytes, glioma cells, glioma stem cells (GSCs), myeloid-derived suppressor cells (MDSCs), natural killer (NK) cells, dendritic cells (DCs), neutrophilic granulocytes, and astrocytes, all of which interact to create an immunosuppressive and metabolically supportive niche for tumor growth and invasion. Under hypoxic conditions, hypoxia-inducible factor (HIF) orchestrates metabolic reprogramming by enhancing glucose uptake via glucose transporters (GLUT), increasing glycolysis, and converting pyruvate into lactate through lactate dehydrogenase A (LDH-A), with lactate export leading to extracellular acidification. HIF also inhibits mitochondrial oxidative phosphorylation (OXPHOS) by activating pyruvate dehydrogenase kinase 1 (PDK1), thereby blocking pyruvate dehydrogenase (PDH) activity and limiting tricarboxylic acid (TCA) cycle flux. Reactive oxygen species (ROS) produced by mitochondria are tightly regulated through glutathione (GSH) biosynthesis to maintain redox homeostasis. Additionally, hypoxia-driven glutaminolysis supplies glutamate to fuel the TCA cycle and biosynthesis of amino acids and fatty acids, essential for tumor cell proliferation. These metabolic adaptations are reinforced by oncogenic signaling pathways, including c-Myc, Ras, and PI3K/Akt, and the suppression of tumor suppressors such as p53, PTEN, and von Hippel-Lindau protein (pVHL). Together, these processes underscore the metabolic plasticity and immune evasion strategies of glioblastoma cells, contributing to tumor progression and resistance to therapy. HIF, Hypoxia-inducible factor; GLUT, Glucose transporter; LDH-A, Lactate dehydrogenase A; PDK1, Pyruvate dehydrogenase kinase 1; PDH, Pyruvate dehydrogenase; TCA, Tricarboxylic acid cycle; OXPHOS, Oxidative phosphorylation; ROS, Reactive oxygen species; GSH, Glutathione; GSCs, Glioma stem cells; MDSCs, Myeloid-derived suppressor cells; NK cells, Natural killer cells; DCs, Dendritic cells; pVHL, von Hippel-Lindau protein.

The metabolic demands and pathways of glioma cellular subpopulations exhibit significant differences, which are closely linked to their functional states and dynamically regulated by the tumor microenvironment (**Figure 2**). Advances in technologies such as single-cell sequencing have gradually unveiled the metabolic characteristics of these subpopulations, highlighting the complexity of the heterogeneous metabolic network within gliomas. Glioma Stem-like Cells (GSCs) represent a critical subpopulation within gliomas, strongly associated with tumor self-renewal, invasiveness, and therapy resistance (38). Studies have shown that GSCs exhibit remarkable metabolic flexibility, enabling them to adjust their metabolic strategies in response to microenvironmental conditions to meet survival demands (39, 40). The metabolic characteristics of GSCs primarily include fatty acid oxidation (FAO), which serves as the main energy source under hypoxic and nutrient-deprived conditions (41). This pathway provides efficient energy production and biosynthetic precursors to maintain stemness and survival. Under conditions of rapid proliferation or extreme hypoxia, GSCs significantly enhance glycolysis (42). Elevated levels of HIF-1α activate glycolysis-related genes such as HK2 and LDHA, supplying energy and metabolic intermediates essential for tumor growth (21). Furthermore, studies have found that glioblastoma stem cells responsible for tumor growth and drug resistance rely on high activity of malate dehydrogenase 2 (MDH2) to maintain their malignant phenotype. Targeting MDH2 has been shown to effectively inhibit GSC proliferation and self-renewal and suppress tumor growth *in vivo (* 43). These metabolic strategies meet the energy and biosynthetic requirements necessary for rapid proliferation, with glycolysis rapidly generating ATP to sustain high proliferation rates and providing intermediates as precursors for the synthesis of nucleotides, lipids, and other macromolecules. Moreover, the accumulation of lactate further exacerbates the acidic tumor microenvironment, suppressing effector immune cell functions and enhancing immune evasion by upregulating immune checkpoint molecules such as PD-L1 (44). Spatial transcriptomics data have revealed significant spatial distribution differences among glioma myeloid cell subpopulations between the tumor core and peripheral regions. Myeloid cells exhibit distinct distribution patterns based on the degree of hypoxia, with immunosuppressive cells showing higher aggregation in the tumor core (29). In hypoxic conditions, TAMs express high levels of immune checkpoint molecules such as PD-L1/PD-L2, significantly suppressing T-cell activation and promoting immune evasion. These findings underscore the metabolic and spatial adaptations of GSCs and tumor-associated cells, offering potential therapeutic targets for glioblastoma treatment.

### 3.2 Dynamics of metabolic changes

The metabolic characteristics of glioma cellular subpopulations are significantly influenced by the dynamic changes in the tumor microenvironment. Glycolytic pathways are enriched in hypoxic core regions and hypoxia-associated areas (37). Immune-reactive regions exhibit a significant accumulation of myeloid and lymphoid cells. Compared to hypoxic and neurodevelopmental regions, immune-reactive regions show higher levels of PD-1 protein on T cells, suggesting the presence of localized immunosuppression. Hypoxia in the tumor core enhances glycolysis and lactate metabolism through the activation of the HIF-1α pathway, further reinforcing an immunosuppressive state (45). To better understand the global metabolic differences within the tumor hierarchy, comprehensive and unbiased steady-state metabolic profiling has been performed in gliomas (43). GSCs, responsible for tumor growth and drug resistance, rely on the high activity of malate dehydrogenase 2 (MDH2) to maintain their malignant phenotype. MDH2 plays a pivotal role in sustaining GSC metabolic and epitranscriptomic networks, paving the way for the development of more effective targeted therapeutic strategies. Additionally, under conditions of glucose or glutamine deprivation, GSCs demonstrate elevated fatty acid oxidation activity, whereas proliferative cells are significantly inhibited under the same conditions (46). Different glioma subpopulations exhibit distinct metabolic properties. At the protein level, glioblastomas have been classified into two novel subtypes: the immune subtype and the neuro-metabolic subtype (47). Studies have identified dihydropyrimidine dehydrogenase and thymidine phosphorylase as potential prognostic biomarkers associated with nucleotide metabolism reprogramming, with their high expression closely linked to poor prognosis. Furthermore, oxidative stress in microglia induces an NR4A2-regulated transcriptional mechanism to rewire cholesterol metabolism (48). These metabolic characteristics not only highlight the complexity of gliomas but also provide potential directions for developing metabolism-targeted therapies against specific subpopulations. Future studies should integrate single-cell and spatial transcriptomics to further explore the critical role of metabolic reprogramming in glioma progression and therapy.

## 4 Applications of single-cell technologies in glioma metabolism research

The emergence of single-cell technologies has provided a novel perspective for studying glioma metabolism. By analyzing gene expression and metabolic activities at single-cell resolution, these technologies have not only unveiled the heterogeneity of metabolic reprogramming in gliomas but also explored the interactions between cells and their microenvironment as well as the dynamic regulation of metabolism. Below are the widely applied single-cell technologies in glioma metabolism research and their specific contributions.

Single-cell technologies have been pivotal in characterizing the immune landscape of gliomas, helping to identify novel, targetable molecular mechanisms that could inform new therapeutic approaches for glioblastoma (GBM). Single-cell sequencing of the GBM tumor immune microenvironment (TIME) has revealed that microglia experience severe oxidative stress, which induces NR4A2-dependent transcriptional activity within these cells (48). Furthermore, spatially resolved transcriptomic analyses of monocyte-derived tumor-associated macrophages (Mo-TAMs) in 51 patients with IDH wild-type glioblastoma or IDH-mutant gliomas have revealed that hypoxic TAMs exhibit increased proportions and adrenomedullin expression levels (34). These features are associated with high vascular permeability and poor prognosis. Single-cell RNA sequencing (scRNA-seq) of patient-derived xenografts and patient samples has shown that astrocyte-like and mesenchymal-like glioblastoma cells exhibit the highest connectivity scores within the tumor microenvironment. Using single-cell and multi-omics analyses, researchers have identified pro-tumor metabolic reprogramming within specific TAM subpopulations (36). For instance, lipid-laden macrophages, characterized by epigenetic reprogramming and immunosuppressive features, are enriched in mesenchymal glioblastoma. These macrophages acquire cholesterol by engulfing myelin debris and directly transferring lipids to cancer cells, supporting the metabolic demands of the tumor.

Spatial transcriptomics has proven capable of distinguishing the metabolic characteristics of gliomas. Researchers analyzing the spatial expression profiles of migration-related genes found an approximate negative correlation between the expression of hypoxia response genes and migration-related genes at the spatial level (37). Mesenchymal-like tumor cells undergo phenotypic reprogramming, which can reshape their surrounding microenvironment, with dysfunctional T cells accumulating in mesenchymal-like regions of glioma tissues. Additionally, fluorescence-guided neurosurgical resection using 5-aminolevulinic acid (5-ALA) can visualize invasive tumor regions beyond the MRI-enhanced area during surgery. The invasive 5-ALA+ cell population exhibits transcriptional similarity with GBM and myeloid cells, characterized by mesenchymal subtypes, active wound response, and glycolytic metabolic features. These cells are located at the invasive margins, in contrast to the tumor core (49).

Single-cell RNA sequencing, spatial transcriptomics, and advanced metabolic analysis tools have demonstrated powerful resolution capabilities in glioma metabolism research. From single-cell resolution gene expression to the spatial dimension of metabolic features, coupled with predictive tools for quantifying metabolic activity, these technologies provide critical insights into the mechanisms underlying glioma metabolic reprogramming. Furthermore, they offer new strategic directions for precise therapies targeting metabolic pathways. Future research should focus on integrating these technologies to support a deeper exploration of heterogeneous metabolic networks in gliomas, paving the way for novel therapeutic approaches.

## 5 Metabolic targeted therapy for gliomas

Metabolic reprogramming in gliomas provides crucial support for their rapid proliferation, invasiveness, and treatment resistance, making the targeting of metabolic pathways an important area of glioma therapy research. Current studies primarily focus on developing targets in glutamine metabolism, glycolysis, and lipid metabolism (50, 51). Glutaminase (GLS) inhibitors, such as CB-839, have demonstrated significant anti-tumor effects in preclinical models by inhibiting glutamine metabolism and disrupting the carbon and nitrogen supply essential for tumor cell survival (51). Key enzymes in the glycolytic pathway, including lactate dehydrogenase A (LDHA) and hexokinase 2 (HK2), also represent potential therapeutic targets. Inhibiting these enzymes reduces lactate production, improves the acidic tumor microenvironment, and enhances immune function (42). Additionally, strategies targeting fatty acid oxidation (FAO) have shown promise in anti-tumor therapy by disrupting the energy supply for glioma stem-like cells (52). Due to the limitations of single-agent metabolic targeted therapies—caused by pathway redundancy and tumor plasticity—combination therapy has emerged as a promising approach. For instance, combining metabolic inhibitors with immune checkpoint inhibitors (e.g., anti-PD-1/PD-L1 antibodies) can reduce lactate accumulation, alleviating immunosuppression and enhancing tumor responsiveness to immunotherapy (48, 53). Similarly, combining metabolic inhibitors with radiotherapy or chemotherapy can weaken tumor energy metabolism and antioxidant capacity, thereby improving treatment sensitivity and overcoming resistance (42).

Overall, metabolic targeted therapy shows great promise in glioma research and clinical applications. Future advancements will require integrating single-cell technologies and multi-omics data to unravel the complexities of glioma metabolic networks. This will drive the rapid development of precision medicine and personalized treatment strategies, offering new solutions for the treatment of refractory gliomas.

## 6 Challenges and future directions

Despite significant progress in glioma research using single-cell technologies and metabolomics, several technical challenges remain. One of the primary difficulties is the integration of high-dimensional single-cell data with metabolomics data due to discrepancies in resolution, time, and spatial dimensions. Additionally, most current studies focus on static analyses, lacking a comprehensive investigation into the dynamic temporal and spatial changes of tumor metabolic characteristics. Particularly in spatial transcriptomics, limitations in the ability to capture and resolve low-abundance metabolic molecules hinder a full understanding of the relationship between metabolism and spatial distribution, presenting a critical bottleneck for precise metabolic research.

In clinical translation, targeted therapies against metabolic pathways face challenges in verifying specificity and safety. Glioma metabolic pathways exhibit high redundancy, and many targets are widely expressed in normal tissues, increasing the risk of treatment-related toxicity. Moreover, clinical trials targeting metabolic pathways often involve small sample sizes and lack long-term follow-up data, limiting comprehensive evaluation of therapeutic efficacy and safety. Identifying patients suitable for metabolic-targeted therapies and conducting large-scale, multi-center clinical trials are crucial for translating these strategies into clinical practice.

Future research should focus on spatiotemporal dynamic analyses of metabolic states to capture how tumor metabolism evolves during different stages of disease progression or before and after treatment. Leveraging spatiotemporal dynamic data from single-cell technologies to study the metabolic reprogramming processes in gliomas will provide theoretical support for developing dynamic precision therapy strategies. Furthermore, the complex interactions between glioma metabolism and the immune microenvironment require deeper exploration. Specifically, the regulatory mechanisms of lactate metabolism on the immunosuppressive microenvironment and the role of lipid metabolism in modulating the functions of TAMs are areas of high interest. These studies could offer new insights for developing combined metabolic and immune-based therapeutic approaches.

Technological integration is another critical future direction. Integrating single-cell transcriptomics, metabolomics, proteomics, and other multi-omics data has the potential to reveal the molecular mechanisms underlying metabolic reprogramming in gliomas and identify novel therapeutic targets. Large-scale integrative studies will further drive the development of precision medicine and provide foundational support for the clinical application of glioma metabolic targeted therapies.

## 7 Conclusion

The application of single-cell sequencing technologies has played a pivotal role in uncovering the cellular heterogeneity and metabolic characteristics of gliomas. Through high-resolution gene expression analysis, researchers have revealed significant differences in the metabolic demands and functions of various cellular subpopulations. By integrating spatial transcriptomics and other advanced technologies, the metabolic distribution patterns between tumor core and peripheral regions have been comprehensively elucidated. The combined application of these technologies provides a novel perspective for understanding the complex biological behavior of gliomas and establishes a solid foundation for exploring metabolism-related therapeutic strategies. Future research should focus on integrating single-cell technologies, multi-omics data, and spatiotemporal dynamic analyses to uncover critical nodes of metabolic reprogramming during tumor progression. Additionally, an in-depth exploration of the interactions between metabolism and the immune microenvironment will offer new insights into the development of combined metabolic and immune-based therapies. By optimizing the specificity and safety of metabolic targets and developing personalized treatment strategies based on patients’ metabolic profiles, there is great potential for achieving revolutionary breakthroughs in precision medicine for glioma treatment.
